# Competing definitions of contextual environments

**DOI:** 10.1186/1476-072X-5-55

**Published:** 2006-12-07

**Authors:** Zaria Tatalovich, John P Wilson, Joel E Milam, Michael Jerrett, Rob McConnell

**Affiliations:** 1 Department of Geography, University of Southern California, Los Angeles, California 90089-0255, USA; 2 Department of Preventive Medicine, University of Southern California; Los Angeles, California 90033, USA

## Abstract

**Background:**

The growing interest in the effects of contextual environments on health outcomes has focused attention on the strengths and weaknesses of alternate contextual unit definitions for use in multilevel analysis. The present research examined three methods to define contextual units for a sample of children already enrolled in a respiratory health study. The Inclusive Equal Weights Method (M1) and Inclusive Sample Weighted Method (M2) defined communities using the boundaries of the census blocks that incorporated the residences of the CHS participants, except that the former estimated socio-demographic variables by averaging the census block data within each community, while the latter used weighted proportion of CHS participants per block. The Minimum Bounding Rectangle Method (M3) generated minimum bounding rectangles that included 95% of the CHS participants and produced estimates of census variables using the weighted proportion of each block within these rectangles. GIS was used to map the locations of study participants, define the boundaries of the communities where study participants reside, and compute estimates of socio-demographic variables. The sensitivity of census variable estimates to the choice of community boundaries and weights was assessed using standard tests of significance.

**Results:**

The estimates of contextual variables vary significantly depending on the choice of neighborhood boundaries and weights. The choice of boundaries therefore shapes the community profile and the relationships between its components (variables).

**Conclusion:**

Multilevel analysis concerned with the effects of contextual environments on health requires careful consideration of what constitutes a contextual unit for a given study sample, because the alternate definitions may have differential impact on the results. The three alternative methods used in this research all carry some subjectivity, which is embedded in the decision as to what constitutes the boundaries of the communities. The Minimum Bounding Rectangle was preferred because it focused attention on the most frequently used spaces and it controlled potential aggregation problems. There is a need to further examine the validity of different methods proposed here. Given that no method is likely to capture the full complexity of human-environment interactions, we would need baseline data describing people's daily activity patterns along with expert knowledge of the area to evaluate our neighborhood units.

## Background

In recent years, health researchers have increasingly emphasized the importance of contextual environments for understanding individual health outcomes [1-6]. These types of studies are typically referred to as *contextual analysis *[7] and they usually involve one or more forms of *multilevel analysis*. The principles of multilevel analysis have been described in detail by numerous authors [8-11]. Essentially, multilevel analysis uses data sets on individuals nested within neighborhoods and enables simultaneous examination of the effects of individual and group level variables on individual level outcomes [11]. The logic behind incorporating group level data into analysis is that they may provide some additional information that cannot be adequately examined or measured at the individual level. This aspect makes multilevel analysis appealing for a variety of topical areas, ranging from violent crime to air pollution [12-17].

Multilevel modeling typically relies on the use of fixed administrative units to define contextual units (e.g. communities, neighborhoods) [9], but there is a concern that this approach may bias the results because the administrative units do not always correspond to the relevant areas in the analysis, and may be crude and incomplete proxies for the characteristics of neighborhoods that affect individual health and behavior. The major dissatisfaction with this approach stems from the well-known Modifiable Areal Unit Problem (MAUP), whereby data and relationships between data can be influenced by the size and shape of units in which data are reported [18-24].

There is an emerging interest to define neighborhoods with greater precision. This trend is likely due to the increase in the availability of social data coupled with the fact that Geographic Information Systems (GIS) offer increasing capabilities for the collection, management, analysis, and representation of geographically referenced social data [25]. While in the past researchers had to use whatever data for whatever zones were available and the units of analysis were regarded as fixed, today they have an opportunity to design a zoning system that is considered best for the particular research purpose [26]. There are more opportunities to go around, to account for, minimize, or otherwise handle the potential aggregation problems that arise with the use of socially referenced data.

For example, Cockings and Martin [27] used zone design techniques, originally developed by Openshaw and Rao [28], to create zones with more stable estimates of socio-economic variables using pre-aggregated data, and explored the sensitivity of statistical relationships between the socioeconomic variables and disease prevalence to changes in zoning system.

Another approach used local spatial statistics with substantive health knowledge to define distinct neighborhoods based on known determinants of health. This approach is exemplified by the work of Jerrett et al. [29], Finkelstein et al. [30] and Jerrett and Finkelstein [31].

The Project on Human Development in Chicago Neighborhoods (PHDCN) [32] defined neighborhoods by collapsing 847 census tracts in the city of Chicago to form 343 neighborhood clusters of approximately the same population size that were internally homogenous on key census indicators. Pre-existing knowledge of Chicago Neighborhood guided this process.

Although methodologically different, the aforementioned procedures have all used homogeneity of census indicators as basic criteria to distinguish communities. Once this was achieved, the sample of individuals could be drawn from each respective neighborhood for further multilevel analysis. Often, however, in multilevel research there is a need to define contextual environments for an existing study sample (e.g. groups of children that were sampled from different schools across the city). The principal challenge in this instance is to understand what constitutes the relevant neighborhood contexts for different groups of subjects.

Recent approaches include 1) the derivation of some functional unit (e.g. a buffer) around places of interest (e.g. individual houses) to define the contextual environment, and 2) transforming the context into some continuous field to assign context as a function of distance decay away from the individual observation. The first approach is exemplified by the work of Frank [33] where 0.5 km buffers were generated around the subject's home to define the likely neighborhood and landscape influences on health. For the second approach, Chaix et al. [34] have developed a model for spatial assignment of neighborhood characteristics in circular areas of constant population size, rather than constant geographic size, thereby generating 'spatially adaptive areas' (areas with a variable window width) of greater size in sparsely populated areas. This approach was inspired by the spatially adaptive filters used in health geography to obtain smoothed maps of disease incidence [35,36].

The use of alternative methods for defining neighborhoods raises a concern regarding the possibility that different routines if applied in the same research context may lead to different conclusions about the neighborhood influence on health. There is a need and an opportunity to experiment with alternative methods for defining neighborhoods and to examine their potential differential impact on the estimates of contextual characteristics.

The present research compared three methods of defining contextual units for the sample of 5,500 children enrolled in the Children's Health Study (CHS). CHS is a longitudinal cohort study focused on the respiratory health of children in 12 Southern California communities [37,38]. This particular study seeks to understand the large differences in rates of childhood asthma between communities that have yet to be explained. Previous research has demonstrated that community characteristics, such as population density, unemployment level and crime, are associated with the amount of stress experienced by individuals as well as poor health outcomes [39]. This suggests that neighborhood characteristics can create unhealthy environments and that large differences between communities in rates of asthma could perhaps be explained by certain contextual characteristics.

The objective of current research is to examine the differential impact of using alternative methods on the estimates of contextual characteristics for the CHS subjects. GIS was used to map the locations of study participants, implement three different approaches for defining neighborhoods, generate estimates of socio-economic variables, examine geographical representation of the relationships between the variable estimates across different communities, and to indicate the locations where these estimates vary significantly. The sensitivity of variable estimates to the choice of boundaries was tested using standard tests of significance.

## Results

### Community boundaries

**Figure 1:**
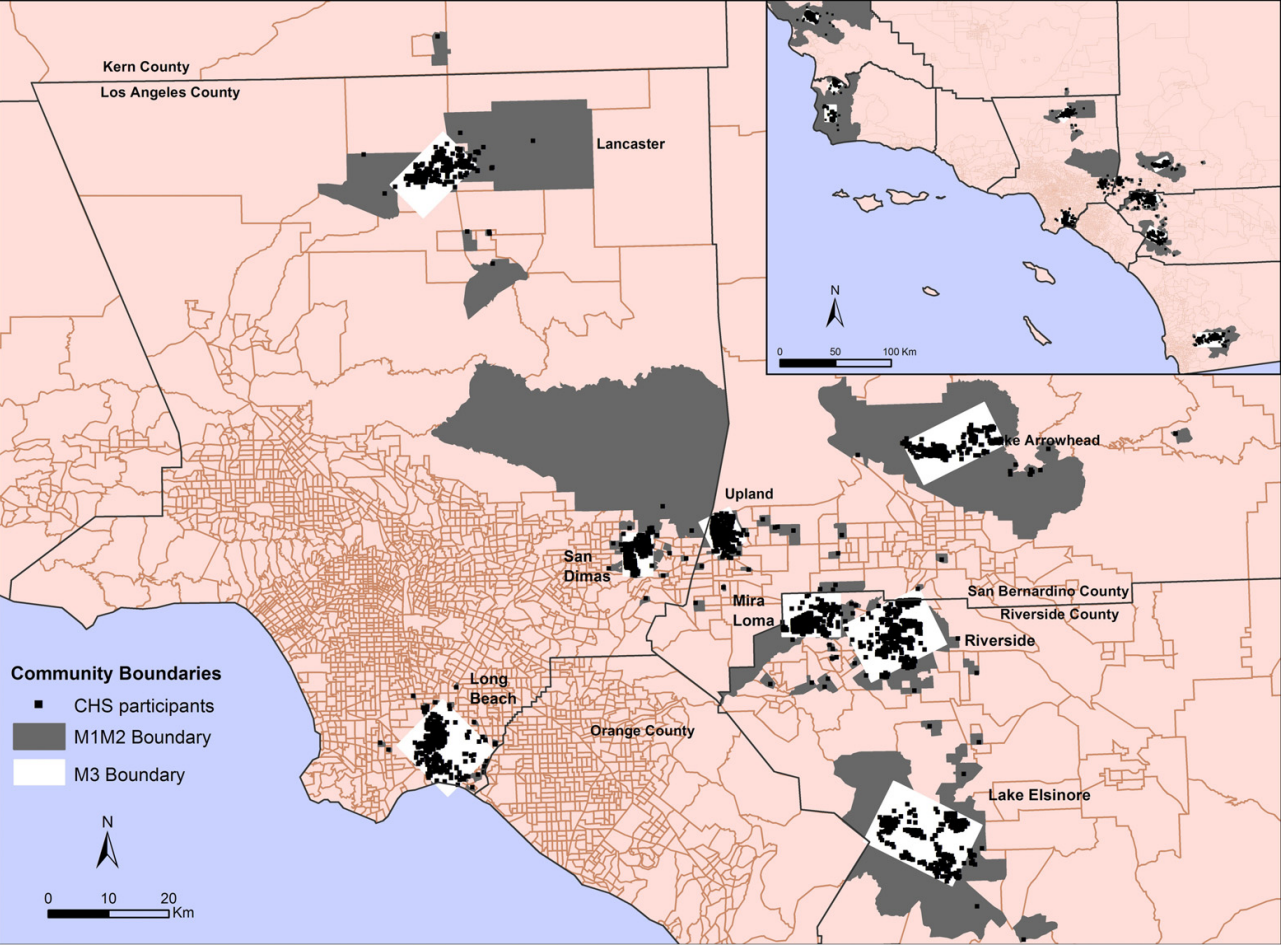
Community boundaries generated by Inclusive Equal Weights (M1), Inclusive Sample Weighted (M2), and Minimum Bounding Rectangle (M3) methods for sample of CHS communities.

**Figure 2:**
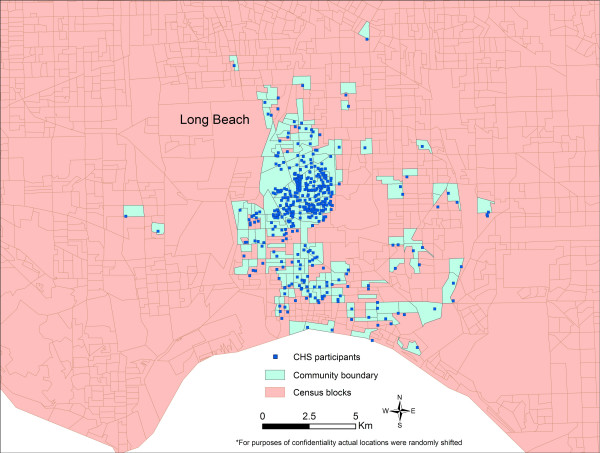
Long Beach community of CHS participants defined using Inclusive Equal Weights (M1) and Inclusive Sample Weighted (M2) methods.

The boundaries of the 12 communities generated by the Inclusive Equal Weights (M1) and Inclusive Sample Weighted (M2) methods generally occupy larger geographic areas than the boundaries produced by the Minimum Bounding Rectangle method (M3) (Figure 1). Long Beach did not follow this pattern because the census blocks with residences of CHS participants are fairly small, reflecting the high densities prevalent in this highly urbanized area, and because the census blocks with CHS participants in Long Beach are, for the most part, not connected so the community boundary generated by M1 and M2 is composed of numerous disconnected census blocks (Figure 2).

### The differences between variable estimates

**Figure 3:**
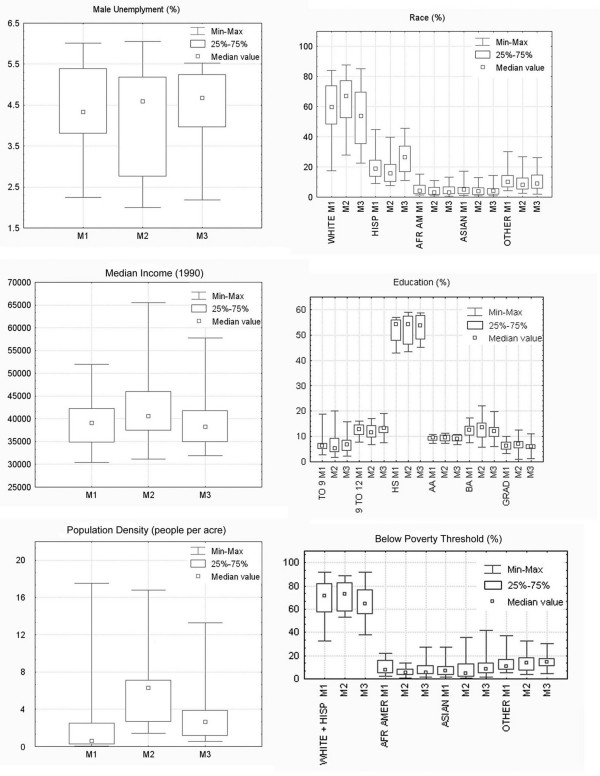
Box-plots representing distributions of variable estimates generated by Inclusive Equal Weights (M1), Inclusive Sample Weighted (M2), and Minimum Bounding Rectangle (M3) methods across 12 CHS communities.

The "box-plots" used in exploratory data analysis reveal the differences in the distribution of community variable estimates generated by these three methods (Figure 3). The first box-plot represents the distributions of estimates of male unemployment. It is evident that M1 and M2 generated distributions with similar minimum and maximum values; however, the inter-quartile range occupied by the middle 50% of values is greater for estimates produced with M2. The median is lowest in M1, and very similar for the M2 and M3 distributions.

The box-plot of median household income is quite different; M1 and M3 produced comparable distributions, while the M2 distribution differs considerably in terms of minimum, maximum and median values as well as inter-quartile range.

The distributions of estimates of population density vary between the three methods in yet, another way. In this instance, M1 and M3 produced similar minimum values, while M1 and M2 generated comparable maximum values. The medians and inter-quartile ranges are noticeably different between all three methods.

The estimates of population proportions in the different racial, educational, and income (i.e. poverty) categories vary between the methods for some categories more than others. For example, the distributions of White and Hispanic population proportions seem to vary more substantially than the distributions across the other racial/ethnic categories, in part because these two groups were the most prevalent across the 12 CHS communities.

The results of the Wilcoxon Matched Pairs Test (Table 1) confirm the general findings of the exploratory data analysis. The differences in the estimates of unemployed males between the three methods are not statistically significant. In contrast, the estimates of median household income are significantly different between M1 and M2, and between M2 and M3 using a 10% level of significance. Similarly, the estimates of population density are significantly different between M1 and M2, and between M2 and M3 (p < 0.05).

**Table 1:** Wilcoxon test of differences in variable estimates between methods

	Wilcoxon Test			
Variable	Pair of Methods	Valid N	z	p-level

UNEMPLOYMENT	M1/M2	12	1.18	0.240
	M1/M3	12	0.00	1.000
	M2/M3	12	1.26	0.210
INCOME	M1/M2	12	2.51	**0.090**
	M1/M3	12	0.24	0.810
	M2/M3	12	2.56	**0.009**
POP DENSITY	M1/M2	12	2.82	**0.004**
	M1/M3	12	1.41	0.150
	M2/M3	12	3.06	**0.002**
RACE	M1/M2	12	2.43	**0.015**
White	M1/M3	12	1.17	0.239
	M2/M3	12	2.98	**0.002**
Hispanic	M1/M2	12	2.12	**0.034**
	M1/M3	12	2.74	**0.006**
	M2/M3	12	3.06	**0.002**
African American	M1/M2	12	2.51	**0.012**
	M1/M3	12	0.88	0.060
	M2/M3	12	1.17	0.239
Asian	M1/M2	12	1.57	0.117
	M1/M3	12	0.88	0.059
	M2/M3	12	0.39	0.695
Oher	M1/M2	12	1.73	0.084
	M1/M3	12	0.94	0.346
	M2/M3	12	1.18	0.239
BELOW POVERTY	M1/M2	12	0.47	0.637
White	M1/M3	12	1.02	0.308
	M2/M3	12	1.25	0.209
African American	M1/M2	12	2.11	**0.034**
	M1/M3	12	0.78	0.432
	M2/M3	12	1.56	0.116
Asian	M1/M2	12	0.87	0.388
	M1/M3	12	0.86	0.388
	M2/M3	12	1.25	0.209
Oher	M1/M2	12	0.00	1.000
	M1/M3	12	1.49	0.136
	M2/M3	12	0.07	0.937
EDUCATION	M1/M2	12	0.94	0.35
To 9 grade	M1/M3	12	0.24	0.813
	M2/M3	12	1.18	0.239
9–12 grade	M1/M2	12	1.33	0.182
	M1/M3	12	0.24	0.813
	M2/M3	12	2.04	**0.041**
HS	M1/M2	12	1.02	0.307
	M1/M3	12	2.51	**0.012**
	M2/M3	12	1.49	0.136
AA	M1/M2	12	1.25	0.209
	M1/M3	12	0.63	0.530
	M2/M3	12	2.35	**0.018**
BA	M1/M2	12	0.63	0.530
	M1/M3	12	0.86	0.388
	M2/M3	12	1.88	0.059
Graduate	M1/M2	12	1.49	0.136
	M1/M3	12	1.17	0.239
	M2/M3	12	2.11	**0.034**

For the variables recording the population proportions in the various racial/ethnic categories, significantly different estimates were generated by M1 and M2 for the percentage White, Hispanic, and African American population, by M2 and M3 for the percentage White and Hispanic population, and by M1 and M3 for the percentage Hispanic population.

The estimates of households below the federal poverty threshold were statistically different in just one case – M1 and M2 for African Americans. For the variables measuring different levels of educational attainment, M2 and M3 produced significantly different estimates in categories grades 9–12 and AA degrees, while M1 and M3 generated significantly different estimates for the proportion of the population with high school diplomas.

In summary, the Inclusive Equal Weights (M1) and Inclusive Sample Weighted (M2) methods generated significantly different estimates in six of 17 possible cases. Similarly, the Inclusive Sample Weighted (M2) and Minimum Bounding Rectangle (M3) methods produced significantly different estimates in seven cases. In contrast, the Inclusive Equal Weights (M1) and Minimum Bounding Rectangle (M3) methods produced significantly different estimates in only two instances.

The aforementioned results have been supplemented by the geographical representation of the variables whose estimates proved to vary significantly depending on the choice of community definition. This step is useful for understanding the relationship between the variable estimates across the communities, and also for identifying communities in which the difference is so great to produce statistically significant differences.

**Figure 4:**
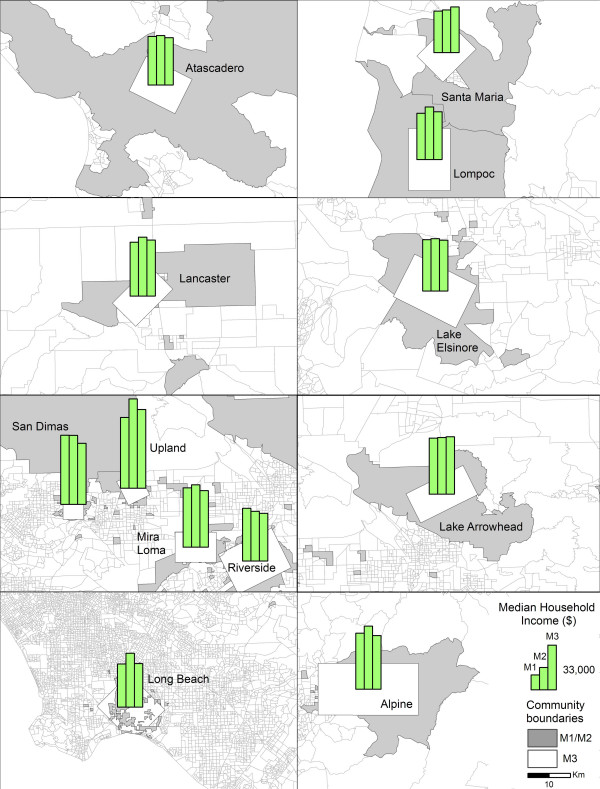
Median household income estimates generated by Inclusive Equal Weights (M1), Inclusive Sample Weighted (M2), and Minimum Bounding Rectangle (M3) methods across 12 CHS communities.

Hence, Figure 4 illustrates the geographical distribution of median household income across the 12 communities, as generated by the three procedures and shows that the Inclusive Sample Weighted Method (M2) produced the highest median household income values in nine out of twelve communities. In Santa Maria and Lake Arrowhead the Minimum Bounding Rectangle Method (M3) generated the highest estimates, while in Riverside, the same is true for the Inclusive Equal Weights Method (M3). Regardless of method, however, these patterns suggest that on an average, Upland has the highest median household income, while Santa Maria has the lowest.

**Figure 5:**
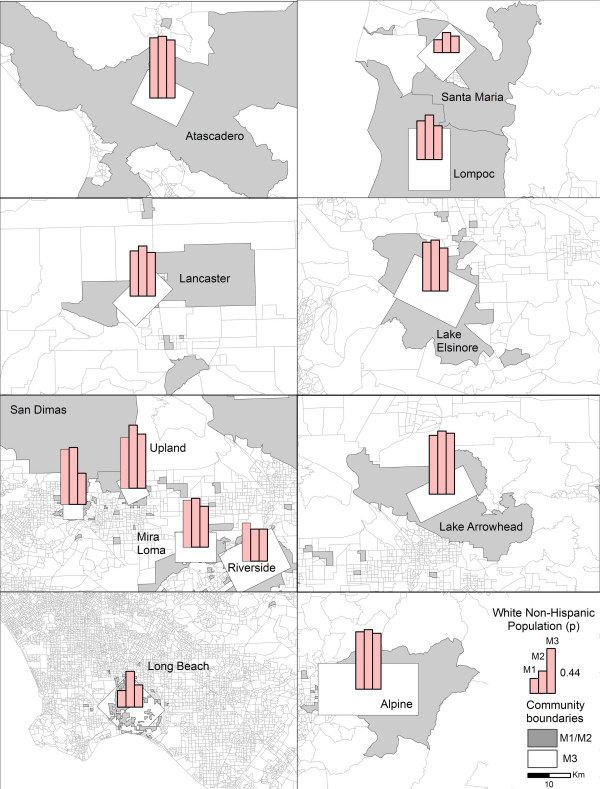
Estimates of White population proportions generated by Inclusive Equal Weights (M1), Inclusive Sample Weighted (M2), and Minimum Bounding Rectangle (M3) methods across 12 CHS communities.

Figure 5 shows the distribution of the White population proportions. In this instance, M2 produced the highest estimates in all 12 CHS communities. The largest differences are evident in Long Beach and San Dimas. At the same time, it appears that M3 significantly underestimated the proportion of White population in San Dimas, in comparison with the two other methods. As presented here, the largest proportion of White population is located in Lake Arrowhead, and the smallest in Santa Maria.

**Figure 6:**
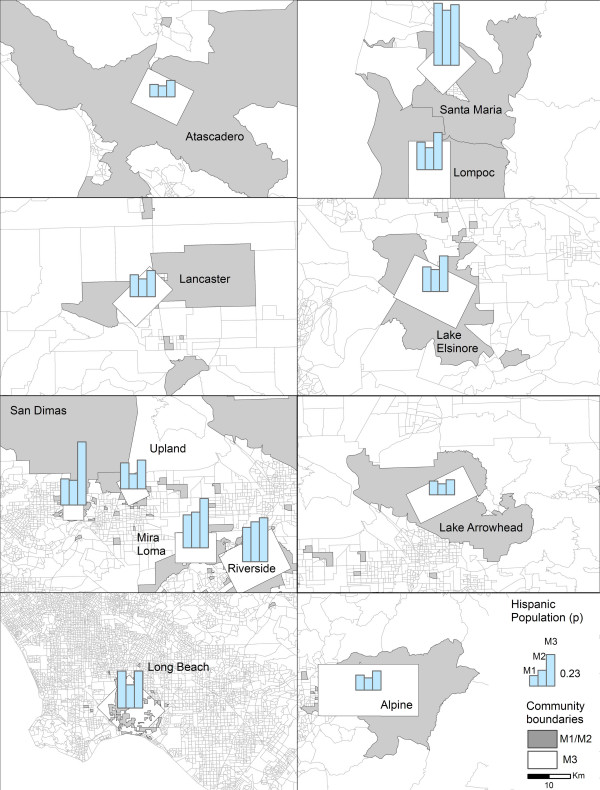
Estimates of Hispanic population proportions generated by Inclusive Equal Weights (M1), Inclusive Sample Weighted (M2), and Minimum Bounding Rectangle (M3) methods across 12 CHS communities.

The Hispanic population proportions are summarized for the 12 communities in Figure 6. In this case, M2 generally produced the lowest estimates, while M3 generated the highest. The geographical distribution also reveals that the greatest proportion of Hispanic population resides in Santa Maria, and the lowest proportions were recorded in Lake Arrowhead, Atascadero, and Alpine.

Overall, the geographical representations of the three variable estimates (as illustrated in figures 4 through 6) revealed that the Inclusive Equal Weights Method (M1), for the most part, mimicked the results generated by the Minimum Bounding Rectangle Method (M3), which is in concordance with the results of the previous exploratory analysis.

## Discussion

The growing interest in the effects of contextual environments on health outcomes has focused attention on alternative community definitions for use in multilevel analysis. Typically, the definition of contextual units relies on administrative boundaries. One of the problems with this approach is that in many cases administrative boundaries do not necessarily correspond to the boundaries of the activity spaces and contextual environments under investigation (see Kwan et al. 2003 [40] for a geographical representation of these activity spaces as built from a series of travel diaries). Increasingly, health researchers are realizing the drawbacks of this approach and are using alternative methods to define contextual units with more precision. However, seldom are the effects of differential methods on the estimates of contextual variables systematically examined.

As a part of the general effort to understand the impact of alternative contextual unit definitions on the estimates of census variables, the present research examined three methods to define contextual units for a sample of children already enrolled in a respiratory health study. GIS proved particularly well suited for this type of analysis, to map the locations of study participants, to define the boundaries of the 12 CHS communities, to compute estimates of sociodemographic variables, to display the geographical relationship between the variable estimates, and to indicate the location where these estimates varied significantly.

The results of statistical analysis suggest that estimates of sociodemographic variables are sensitive to the choice of community boundaries and weights. Similarly the analysis of the geographical distribution of variable estimates supports the idea that the choice of boundaries shapes the neighborhood profile and the relationships between its components (variables).

The results from this study indicate that the Inclusive Equal Weights (M1) and Minimum Bounding Rectangle (M3) methods produced similar estimates; however, this should not be used as reason to dismiss the Inclusive Sample Weighted method as less accurate. In other words, similarity in the results generated by any two methods is not a guarantee of greater accuracy of estimates. The three alternative methods used in this research all carry some subjectivity, which is embedded in the decision as to what constitutes the boundaries of the communities.

While the same rules were applied in each method to assign community boundaries and weights the resulting areas in the cases of M1 and M2 varied substantially along with the size of census block groups – which are typically larger in less populated areas, such as Atascadero, and smaller in densely populated areas, such as Long Beach. This may introduce aggregation problems, which arise when data from fairly large areas are used to define the conditions of small contextual units, as would have happened if relatively small but uniform buffers centered on specific residences had been used to delineate the 12 CHS communities discussed in this paper.

The Minimum Bounding Rectangle Method focused attention on the core areas of the communities based on the premise that these are the areas where most of the study participants conducted their daily lives. This approach was an attempt to focus attention on the most frequently used spaces to control (i.e. minimize) potential aggregation and bias problems. The subjectivity in this instance arises from the selection of a 95% bounding rectangle (as opposed to a 90%, 99%, or some other sized rectangle) to define the contextual units.

## Conclusion

Multilevel analysis requires careful consideration of what constitutes a relevant contextual unit for a given study sample, because the alternative definitions may have differential impacts on the results. We have followed up the present research with empirical tests using actual health data and found that the method of classification has an effect on the sign and significance of the variables' impact on asthma prevalence [41].

There is a need to further examine the validity of different methods proposed here. Given that no method is likely to capture the full range of diversity and complexity of human-environment interactions, we would need baseline data describing the locations and character of people's daily activity patterns along with expert knowledge of the area and subjects under investigation to evaluate our neighborhood units and push our work beyond this conundrum.

## Methods

### Data sources

The analyses performed in this research relied on use of 1990 Census Block Group (BG) boundaries for the Southern California region and the corresponding estimates of population density, income, race, male unemployment rates, proportion of households below federal poverty threshold by racial/ethnic category, and educational attainment. BG is the lowest-level geographic entity for which the United States Census Bureau tabulates sample data from a decennial census and generally contains between 300 and 3,000 people, with an optimum size of 1,500 people. These data were available for download from the US Census Bureau website [42]. 1990 Census was chosen because the study sample was recruited at the beginning of that decade.

**Figure 7:**
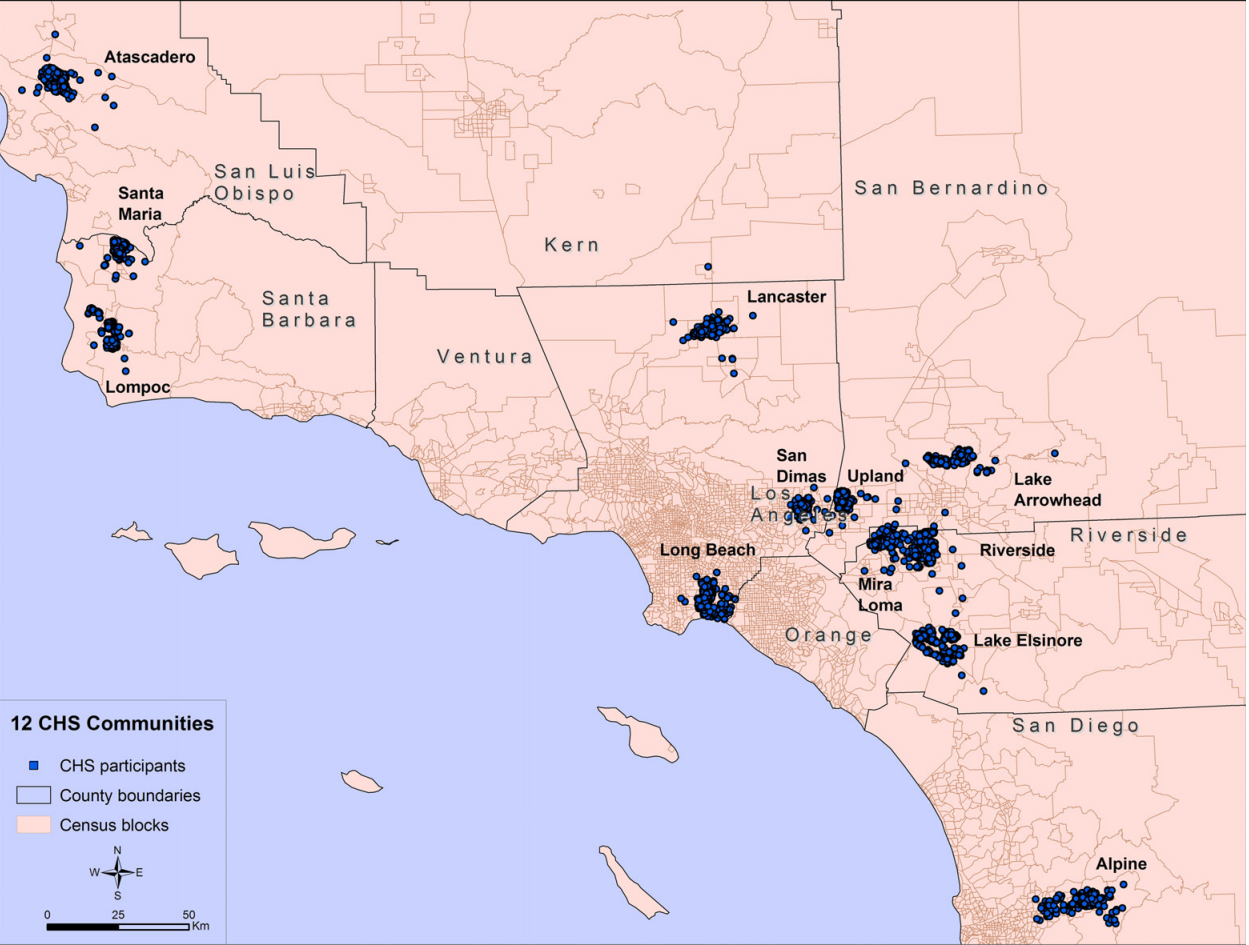
Locations of CHS participants spread across 12 communities.

The Children's Health Study collected information on the location of residences of 5,763 study participants recruited from the 4^th^, 7^th ^and 10^th ^grades in 1993 and 1996, and delivered a baseline table with the following information: unique ID number for each study participant, address, matching X, Y coordinates, and name of community of residence. Study participants were scattered across 12 communities in southern California: Alpine, Atascadero, Lake Arrowhead, Lake Elsinore, Lancaster, Lompoc, Long Beach, Mira Loma, Riverside, San Dimas, Santa Maria, and Upland (Figure 7).

### Base map

At the outset, a base map was generated that showed the distribution of CHS participants within the 12 communities (e.g. cities) (Figure 7). The coordinates of locations of residences of CHS participants were imported to ESRI's ArcGIS (Environmental Systems Research Institute, Inc., Redlands, California) where a point feature layer was created and superimposed over the layer of southern California census block groups. The overlay function in ArcGIS was used to assign census blocks to study participants and thereby link each study participant to their appropriate community.

### Community definitions

Three methods were implemented for defining the areas of the 12 communities with study participants and estimating their sociodemographic characteristics: the Inclusive Equal Weights, Inclusive Sample Weighted, and Minimum Bounding Rectangle Methods. These three methods employed alternate boundary definitions and weights to generate estimates of the six sociodemographic variables noted earlier.

*The Inclusive Equal Weights Method *defined communities using the boundaries of the census block groups that included the households of the sampled children. In estimating community variables all qualifying blocks were assigned equal weights even if they were sampled for the CHS study at different intensities. A sample map for the Long Beach community (Figure 2) shows the boundaries defined using this method. The methods used to estimate community variables are presented below:

Population Density = (P1+...+Pn)/(A1+....+ An)     (1)

where P is the total population of the census block group, A is the area of the block group, and n is the number of block groups in the community. Median household income was computed as:

Median Income = (I1* P1+...+In* Pn)/(P1+...+Pn)     (2)

where I is the median income of the census block group, P is the total population of the block group, and n is the number of blocks within each community. The proportion of people by racial/ethnic category was computed as:

p (racial/ethnic group) = (R*i*1 +...+ R*i*n)/(P1 +...+ Pn)     (3)

where R is number of persons in racial/ethnic category *i *(Hispanic, White, African American, Asian, or Other) per block, P is the total population of the block group, and n is the number of block groups in the community. The estimates generated for the other variables followed this formula.

*The Inclusive Sample Weighted Method *considered the same set of Census blocks as the previous method, but variables were estimated using the weighted proportion of CHS participants per block. In this instance, row census data was first weighted by the proportion of CHS participants within each block, then summed to obtain variable estimates. The community variables were generated as follows:

Population density = P1/A1 * (p)C1 +...+ Pn/An * (p)Cn     (4)

where P is the population of the block group, A is the area of the block group, (p)C is the proportion of CHS participants within a block group, and n is the number of block groups in the community. Median household income was computed as:

Median Income = (I1 * (p)C1 + ... + In * (p)Cn)/(p)C1+... + (p)Cn     (5)

where I is the median household income for the block group, (p)C is the proportion of CHS participants in the block group, and n is the number of block groups. The proportion of people by racial/ethnic category was computed as:

p (racial/ethnic group) = (p) R*i*1 * (p)C1 +...+ (p) R*i*n * (p)Cn     (6)

where (p) R is the proportion of people in racial/ethnic category *i *(Hispanic, White, African American, Asian, or Other) per census block group, (p)C is the proportion of CHS participants per block group, and n is the number of block groups in the community. All other variables follow this example.

**Figure 8:**
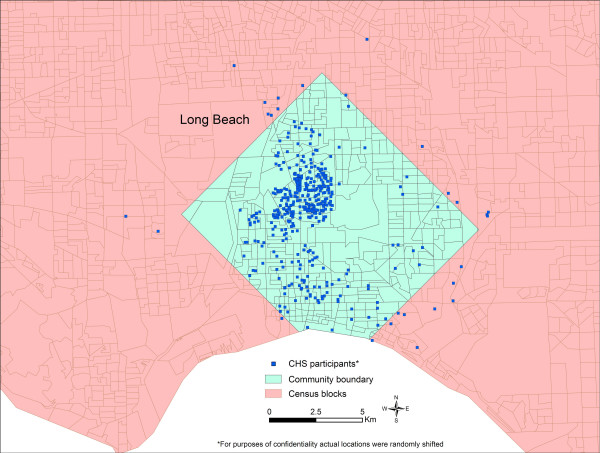
Long Beach community of CHS participants defined using Minimum Bounding Rectangle Method.

*The Minimum Bounding Rectangle Method *defined the boundaries of the communities using minimum bounding rectangles that included 95% of the CHS participants. A sample map of the Long Beach community shows the boundaries defined using this method (Figure 8). As a pattern in all 12 communities, roughly 5% of CHS participants was scattered away from the main cluster, occupying sometimes large and distant census blocks – the rationale for this 95% rectangle method was to help eliminate the influence of these distant block groups when estimating community characteristics. The census variables were estimated using variable estimates of each block group within the 95% rectangles. The community variables were generated as follows:

Population density = (P*r*1+ ...+ P*r*n)/(A*r*1+....+ A*r*n)     (7)

where P*r *is the number of persons within each block group enclosed in the 95% rectangle *r*, and A*r *is area of the block group enclosed in the rectangle *r*. Median household income was computed as:

Median Income = (P*r*1*I1 +...+P*r*(n) * In)/(P*r*1+...+P*r*n)     (8)

where P*r *is the number of people in each block group in the rectangle, I is the median household income of the parent census block group, and n is the number of block groups in the community. The proportion of people by racial/ethnic category was computed as:

p (racial/ethnic group) = (R*i*1 +...+R*i*n)/(P1 +...+ Pn)     (9)

where R*i *is the count of people in racial category *i *(Hispanic, White, African American, Asian, or Other) per census block, P is the total population of the block group, and n is the number of block groups in the community. The estimates generated for the other variables followed this formula.

### Evaluating sensitivity of estimates to the choice of method

Exploratory data analysis was performed at the outset using box-plots to visually examine the distributions of variable estimates generated by the Inclusive Equal Weights (M1), Inclusive Sample Weighted (M2), and Minimum Bounding Rectangle (M3) methods. The box-plot analysis was followed by testing the significance of the differences in estimates between each pair of methods (i.e. M1/M2, M1/M3, and M2/M3) using the Wilcoxon test. This test was used to examine if the estimates of census variables are sensitive to our choice of community boundaries and weights. For each community, the estimates of census variables generated by the three methods were presented on top of a series of base maps to facilitate comparison. The purpose of this evaluation was not to demonstrate that one particular method is better than the other, but rather to examine the variation between estimates depending on the method used.

## Authors' contributions

ZT implemented three alternative methods to define contextual units for study participants, carried out the data analysis andinterpretation of the results, drafted, and revised the manuscript. JPW coordinated the design and implementation of different methods to define contextual units and their socio-demographic characteristics, and helped with revision of the manuscript. JM contributed to the design of an alternative ways to assign weights for estimating community-level socio-demographic variables. MJ helped draft the introduction and contributed to several revisions of the manuscript. RM conceived of the study and participated in its design and coordination.
